# A Human Magnet

**Published:** 1892-11

**Authors:** 


### A HUMAN MAGNET.

Perhaps it is not generally known, but in the city of Lewiston, Me., lives a man who is possessed of a magnetic or some kind of wonderful power which is truly miraculous. Oscar F. Whitman, the man in question, is 36 years old and lives with his wife and little daughter. A reporter called on Mr. Whitman and received a cordial reception. Mr. Whitman is a man about the average height with a pair of broad shoulders and a deep chest. He is in every way an athletic and healthy appearing specimen of humanity, and his face, though at times it appears stern, is in itself a picture of benevolence and kindness.

After a brief conversation the party adjourned to the kitchen and Mr. Whitman proceeded to give an exhibition of his power.

Seating himself at the head of a large table in the middle of the room, Mr. Whitman held his hands out at an angle of about 45 degrees. In a short time a convulsive expression seemed to creep over his face, and placing his finger tips on the table he raised it up and held it in the air as if it was nothing but a sheet of paper. The reporter, with Mr. Whitman's consent, then took a seat at the opposite end of the table and tried to hold it, but the magic finger tips were too much, and he was pulled over chair and all. Now, Mr. Bradbury prides himself on being a pretty strong man, so with a smile on his face he changed his seat for the one at the end of the table. Grasping the table legs firmly he braced his feet, and the look that stole over his face plainly said : "Now, friend spirits or whatever you are, I've got you." He did not have them though, and the 215 pounds of human flesh, chair, and table followed the dictation of the wonderful finger tips over the room in a manner which made every one else laugh.

Mr. Linscott, during these proceedings, had been rubbing up his muscles and preparing for a grip with the supernatural. He took the seat, calmly got a good hold and announced that he was ready. He was ready, too, for his muscles stood out like whipcord, but still he was not "in it," and like the others yielded to the inevitable. Messrs. Tarr and Wood concluded it was no use to try it, and so this part of the proceedings came to an end.

Now some people think that Mr. Whitman is a walking electric plant, but such is not the case, for he placed a pane of glass on the table and placing his fingers on it, lifted it just as if the non-conductor was not there.

Mr. Whitman does not claim to be a spiritualist or an electrician. When he wants to use his latent power he holds his hands out as stated above, at an angle of 45 degrees, and the current starts from his shoulders and runs to his finger tips, and when he is done using it he dips his fingers in cold water, sprinkles a few drops on his forehead, and, holding both hands in the air, allows it to run back to its receptacle in his shoulders. "It seems," says Mr. Whitman, "as if there was a hollow in my bones through which this power is transmitted."

This power was noticed in Mr. Whitman when he was a small boy. Sometimes, when playing with other children, he would place his fingers on a chair and, for his amusement, pull the other children around the room hanging to it. Mr. Whitman grew up the same as other boys, and being of a rather reserved disposition, said little about his powers. He has been working and is now employed as a cutter with the firm of Gay, Woodman & Co., although lately he has not worked more than half the time.

